# Strengths of social ties modulate brain computations for third-party punishment

**DOI:** 10.1038/s41598-023-37286-8

**Published:** 2023-06-28

**Authors:** Zixuan Tang, Chen Qu, Yang Hu, Julien Benistant, Frédéric Moisan, Edmund Derrington, Jean-Claude Dreher

**Affiliations:** 1 Key Laboratory of Brain, Cognition and Education Sciences, Ministry of Education, Guangzhou, China; 2 School of Psychology, Center for Studies of Psychological Application, and Guangdong Key Laboratory of Mental Health and Cognitive Science, South China Normal University, Guangzhou, 510006 China; 3 Laboratory of Neuroeconomics, Institut des Sciences Cognitives Marc Jeannerod, CNRS, 69675 Lyon, France; 4 Université Claude Bernard Lyon 1, 69100 Lyon, France; 5 GATE UMR 5824, EM Lyon Business School, 69130 Ecully, France; 6 Shanghai Key Laboratory of Mental Health and Psychological Crisis Intervention, School of Psychology and Cognitive Science, East China Normal University, Shanghai, 201613 China

**Keywords:** Neuroscience, Psychology

## Abstract

Costly punishment of social norm transgressors by third-parties has been considered as a decisive stage in the evolution of human cooperation. An important facet of social relationship knowledge concerns the strength of the social ties between individuals, as measured by social distance. Yet, it is unclear how the enforcement of social norms is influenced by the social distance between a third-party and a norm violator at the behavioral and the brain system levels. Here, we investigated how social distance between punishers and norm-violators influences third-party punishment. Participants as third-party punished norm violators more severely as social distance between them increased. Using model-based fMRI, we disentangled key computations contributing to third-party punishment: inequity aversion, social distance between participant and norm violator and integration of the cost to punish with these signals. Inequity aversion increased activity in the anterior cingulate cortex and bilateral insula, and processing social distance engaged a bilateral fronto-parietal cortex brain network. These two brain signals and the cost to punish were integrated in a subjective value signal of sanctions that modulated activity in the ventromedial prefrontal cortex. Together, our results reveal the neurocomputational underpinnings of third-party punishment and how social distance modulates enforcement of social norms in humans.

## Introduction

Cooperation among strangers is a major evolutionary puzzle^1,2^. One key mechanism for maintaining cooperation in large groups is that some individuals enforce social norms by applying punishment to defectors^3,4^. In a modified Dictator Game, called the Third-Party Dictator Game (TP-DG)^3,5^, participants as observers may pay to punish greedy dictators that share money unfairly with recipients. Such third-party punishment (TPP) may be costly to the third-party who themselves receive no material benefit^3,6^. Little is known about the brain representations of TPP despite the crucial role it plays in norm enforcement. In contrast, “second-party” punishment (SPP), in which victims retaliate directly against their aggressors, and its underlying brain systems have been thoroughly investigated^5,7^. TPP may be an evolutionary elaboration of this more ancient mechanism^8^. A classical model of SPP defined “egocentric inequity” as the absolute payoff difference between self and others^5,9,10^. This inequity aversion model has been extended to a third-party perspective deciding sanctions based on the inequity between the dictator and the recipient, as perceived by the third-party^11^. However, it is unclear how the strength of the social ties (social distance, SD) between the third-party and the dictator affects the sanctions to the dictator. As SD in a social network determines how much one collaborates with others^12^ and decreases generosity^13,14^, a third-party might be more likely to turn a blind eye when close others violate social norms.

Here we ask how does the strength of social ties alter our tolerance of norm violation? What are the neurocomputational mechanisms engaged in making TPP decisions? To disentangle the role of fairness preferences and SD to the norm violator in punishment, we developed a new TPP computational model and a modified TPP task to investigate how SD affects TPP at both the behavioral and neural level. Participants, as third-party observers, were presented monetary splits advocated by a dictator to a recipient. Crucially, the SD between the third-party (participants) and the dictator varied systematically, but the recipient was always a stranger. Participants were asked how much of their own money they would use to punish the unfair dictators, and the dictator’s payoff would be reduced by three-fold more.

We tested whether TPP increases with higher SD to the norm violator. At the brain level, we sought to identify the neurocomputational mechanisms underpinning the integration between fairness preferences and closeness of social ties with norm violators. A first question was to determine whether brain regions known to represent physical space and more abstract relationships also represent the SD to the norm violator^15^. We hypothesized that a prefronto-parietal system, previously observed in encoding social ties in social networks, would also encode social ties in the context of TPP^16^. Another question was to pinpoint the neurocomputational mechanisms underlying TPP. Indeed, the brain system (anterior cingulate, anterior insula) engaged in secondary-punishment may either reflect social norm concerns or retaliatory motives^3,6^. In contrast, norm enforcement by unaffected third parties cannot be explained by retaliatory motives^17^, therefore our approach has the potential to clearly explain the motives and neurocomputational mechanisms underlying TPP. Finally, a last question was to determine how the brain integrates the different signals of inequity aversion for an unaffected third-party as measured by the payoff difference between the dictator and the recipient), the strength of their social ties to the norm violator, measured by social distance, and the cost of punishing during TPP. Our hypothesis was that the ventromedial prefrontal cortex (vmPFC), which is engaged in evaluating norm violations^11,18^, is performing this integration process when deciding between different levels of punishment.

## Results

### Behavioral results

#### Punishment level

**Figure 1:**
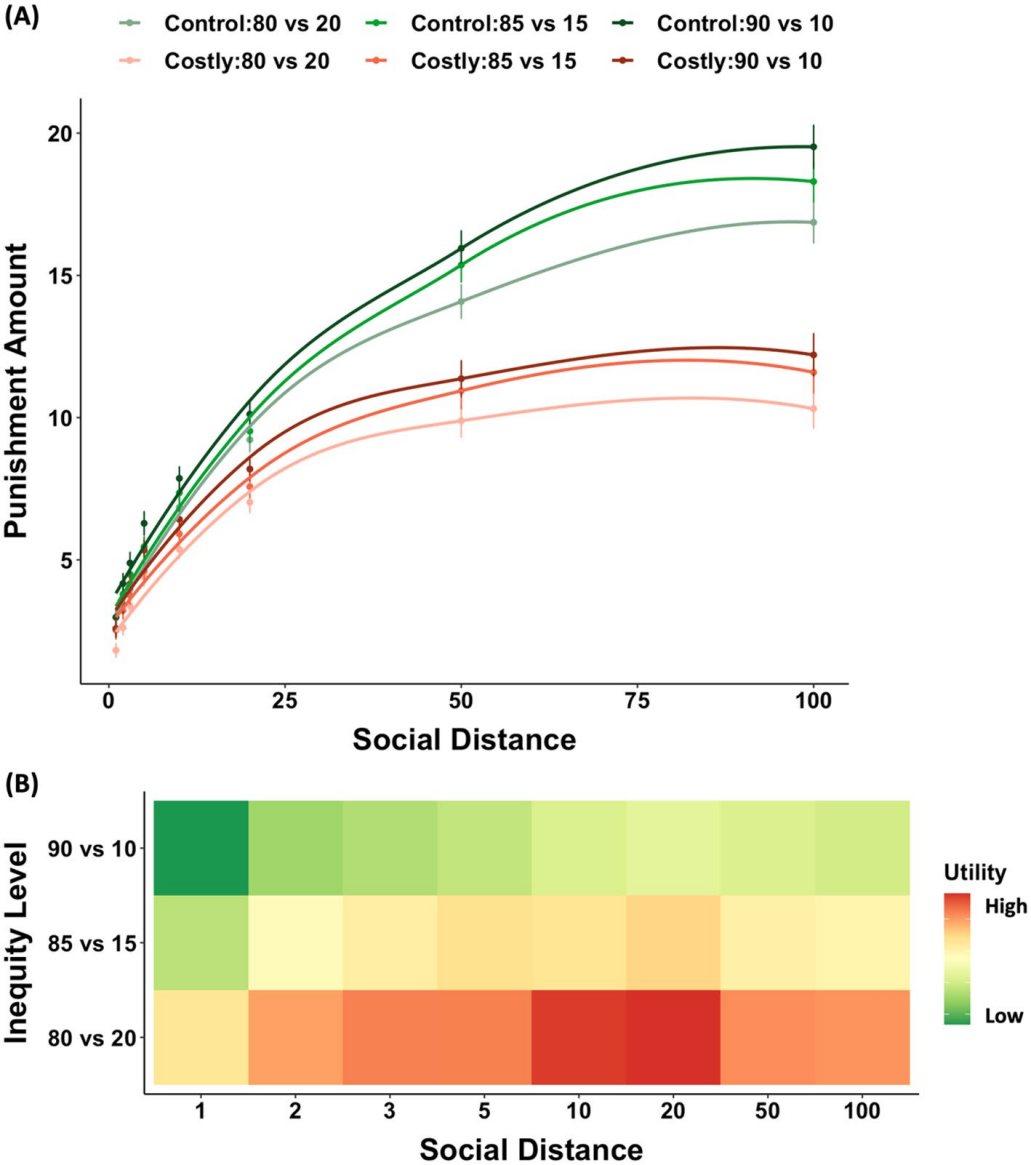
Behavioral results. (**A**) Punishment severity at different social distances. The punishment severity varied as a reversed hyperbolic function of payment condition on social distance (1, 2, 3, 5, 10, 20, 50, 100) for different inequity levels (90 vs. 10, 85 vs. 15, 80 vs. 20) and payment conditions (costly, control). The LOESS (locally weighted scatterplot smoothing) method was used to create smooth trendlines for visualization purpose. The error bars show SEM. (**B**) Variation of utility (measured by Eq. 1) with social distance. The color-coded heatmap shows utility for costly punishment against social distance at each inequity level (red indicates high and green indicates low utility values).

The behavioral choices are shown in Fig. 1A. We studied how payment conditions (Costly/Control), social distances and inequity levels (90 vs. 10/85 vs. 15/80 vs. 20) affected the punishment amount. Participants punished the dictator more severely in the Control (non-costly) condition (8.32 ± 0.74 vs. 6.15 ± 0.72, *F*(1, 30) = 5.94, *p* = 0.021) than the Costly, and when the social distance between the third-party and the dictator was larger (*F*(1, 30) = 50.54, *p* < 0.001), as well as when the unfairness of allocations was greater (*F*(2, 46) = 9.42, *p* < 0.001). The interaction of the three factors was not significant (*F*(2, 64) = 0.95, *p* = 0.392), nor were the interactions between payment conditions and inequity levels (*F*(2, 164) = 0.32, *p* = 0.729), but the interaction between payment conditions and social distances was significant (*F*(1, 30) = 20.57, *p* < 0.001), as was the interaction between social distance and inequity level (*F*(2, 44) = 6.44, *p* = 0.004).

#### Reaction time

We then investigated how payment conditions (Costly/Control), social distances, and inequity levels affected the reaction time. Participants spent more time to make a decision when the social distance increased (*F*(1, 29) = 16.08, *p* < 0.001), but the difference in reaction time between costly and control conditions (*F*(1, 73) = 1.09, *p* = 0.300), and the different inequity levels (*F*(2, 42) = 1.88, *p* = 0.165) were not significant. There were no two-way or three-way interactions between the factors.

### fMRI results

#### Brain systems modulated by social distances

**Figure 2:**
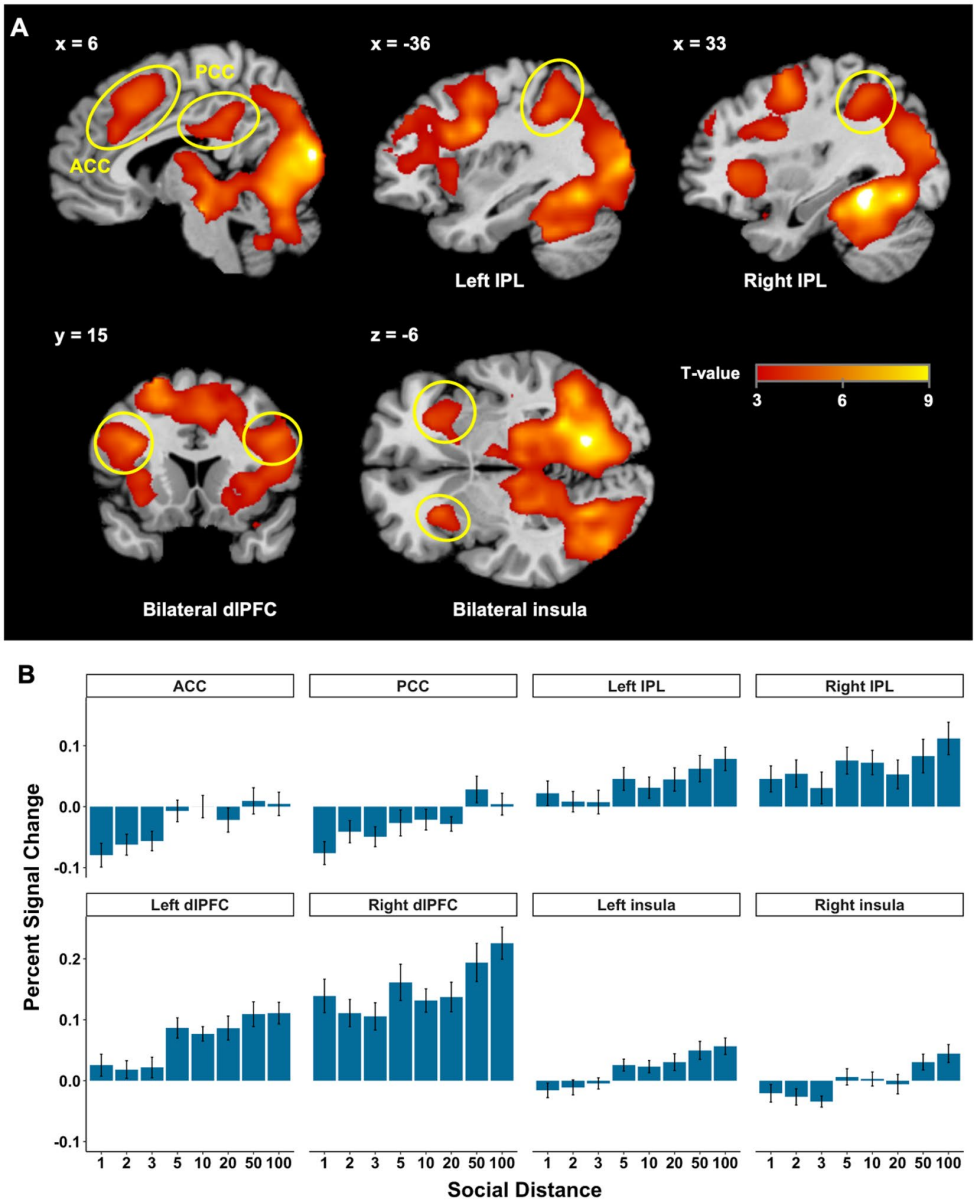
Social distance-related fMRI results. (**A**) Increasing social distance engaged a large bilateral dorsolateral prefrontal cortex-parietal network, including the anterior cingulate cortex (ACC, peak MNI coordinates 6, 30, 27; *t*(30) = 4.46, *p*(SVC–FWE) = 0.008), PCC (peak MNI coordinates − 3, − 33, 30; *t*(30) = 4.93, *p*(SVC–FWE) = 0.002), bilateral IPL (left IPL: peak MNI coordinates − 36, − 42, 39, *t*(30) = 5.69, *p*(SVC–FWE) = 0.001; right IPL: peak MNI coordinates 33, − 48, 45, *t*(30) = 5.08, *p*(SVC–FWE) = 0.005), bilateral dlPFC (right dlPFC: peak MNI coordinates 42, 9, 24, *t*(30) = 6.58, *p*(FWE) = 0.05; left dlPFC: peak MNI coordinates − 36, 15, 24, *t*(30) = 6.37, *p*(FWE) = 0.008), and bilateral insula (left insula: peak MNI coordinates − 27, 21, − 6, *t*(30) = 5.16, *p*(SVC–FWE) = 0.001; right insula: peak MNI coordinates 30, 18, − 9, *t*(30) = 4.30, *p*(SVC–FWE) = 0.007). (**B**) Percent signal changes with increasing social distance in costly third-party punishment. The error bars show SEM.

In a first GLM, we investigated the brain regions engaged with higher inequity levels and with higher social distance during the decision phase (parametric modulators, GLM 1). For SD, both in the Costly and Control (non-costly) punishment conditions, a brain system composed of the bilateral dorsolateral prefrontal cortex (dlPFC), ACC, PCC, bilateral IPL and bilateral insula increased activity with increasing social distance (see Supplemental Material Fig. S1). Since these activations were similar, as SD increased in both the Costly and Control condition, and since costly condition was more meaningful in the context of TPP, we only focused on the effect of costly SD in the rest of this paper. A brain system including bilateral dlPFC, ACC, PCC, bilateral IPL, and bilateral insula, was positively correlated with increasing social distance (see Fig. 2A and Supplemental Material Table S1). To illustrate how the BOLD signal varied with social distance, we extracted the percent signal change from these regions (5 mm radius spheres with center at the reported peak coordinates), and found increased BOLD signal as social distance increased (Fig. 2B).

#### Brain system modulated by inequity levels

**Figure 3:**
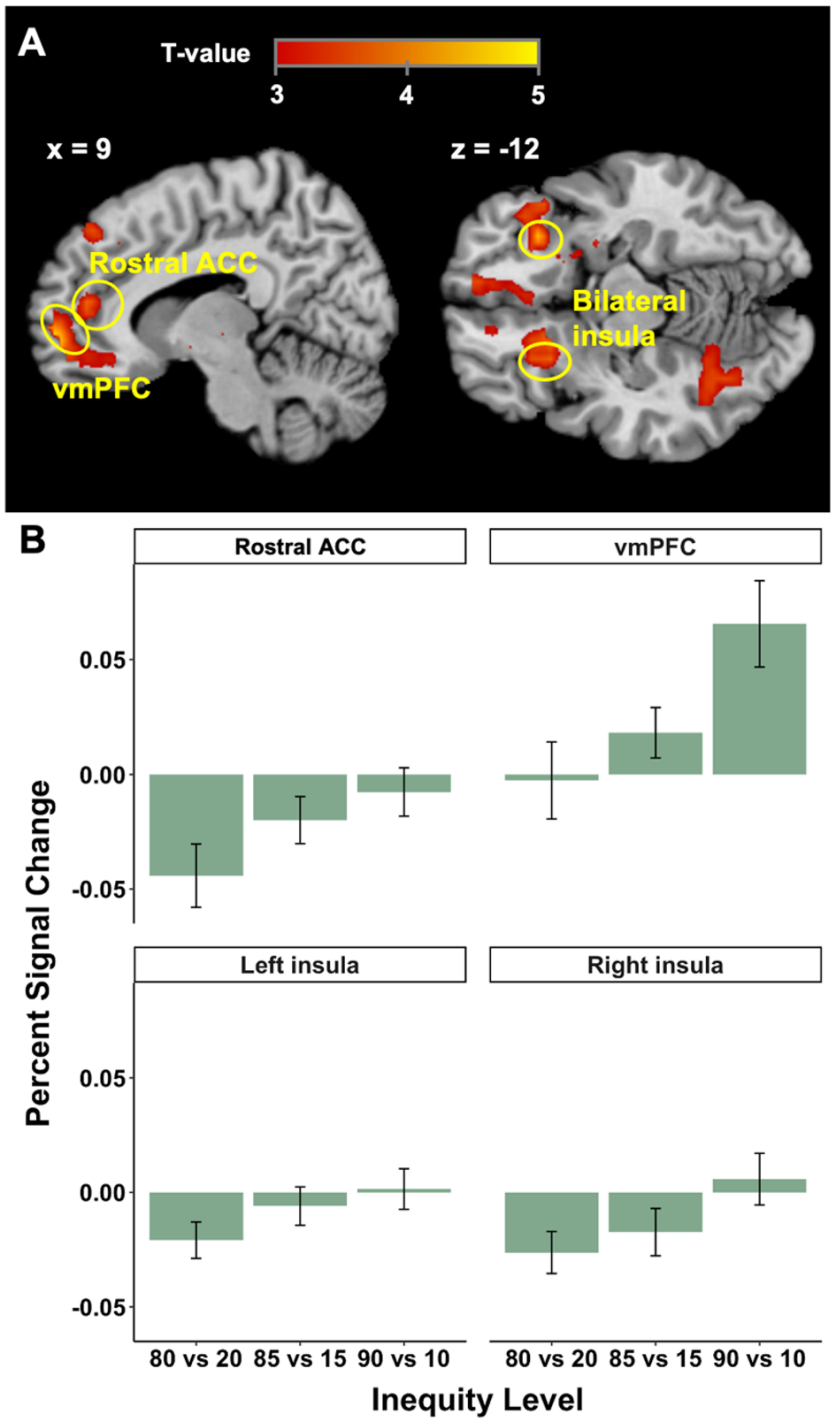
Inequity-related fMRI results (in GLM 1). (**A**) The inequity level was one of the parametric regressors in GLM 1. Rostral ACC (peak MNI coordinates 6, 45, 15; *t*(30) = 3.86, *p*(SVC–FWE) = 0.030), vmPFC (peak MNI coordinates 9, 57, − 6; *t*(30) = 4.43, *p*(SVC–FWE) = 0.005), and bilateral insula (left insula: peak MNI coordinates -24, 18, − 15, *t*(30) = 3.78, *p*(SVC-FWE) = 0.023; right insula: peak MNI coordinates 33, 24, − 15, *t*(30) = 4.43, *p*(SVC–FWE) = 0.005) were positively correlated with increasing inequity level. (**B**) Percent signal changes with increasing inequity level in costly third-party punishment. The error bars show SEM.

Next, we investigated the brain regions in which BOLD signal correlated with inequity level. SPP studies have identified inequity aversion related brain regions in the anterior insula (AI) and rostral anterior cingulate cortex (rACC)^9,19^. We therefore hypothesized these brain regions would also reflect inequity aversion in TPP. As predicted, we found that when inequity levels were higher, the rostral ACC and bilateral insula were more engaged (Fig. 3A). This indicates that these regions are sensitive to unfair allocations in TPP. We also observed engagement of the rostral ACC/vmPFC with higher inequity level. Again, we extracted the percent signal change from the reported activations, and found increased activations at higher inequity levels (Fig. 3B).

#### Brain regions modulated by expected value of the chosen punishment option

Finally, we identified the brain regions encoding the expected value of the chosen punishment option. These regions integrate SD and inequity level to attribute a value that presides the punishment decision. To calculate the expected value of the chosen punishment option, we developed a computational model of the utility of the chosen punishment option (measured by $U({x}_{1},{x}_{2},{x}_{3},{p}_{SD})$ with Eq. (1) in combination with Eq. 2).

**Figure 4:**
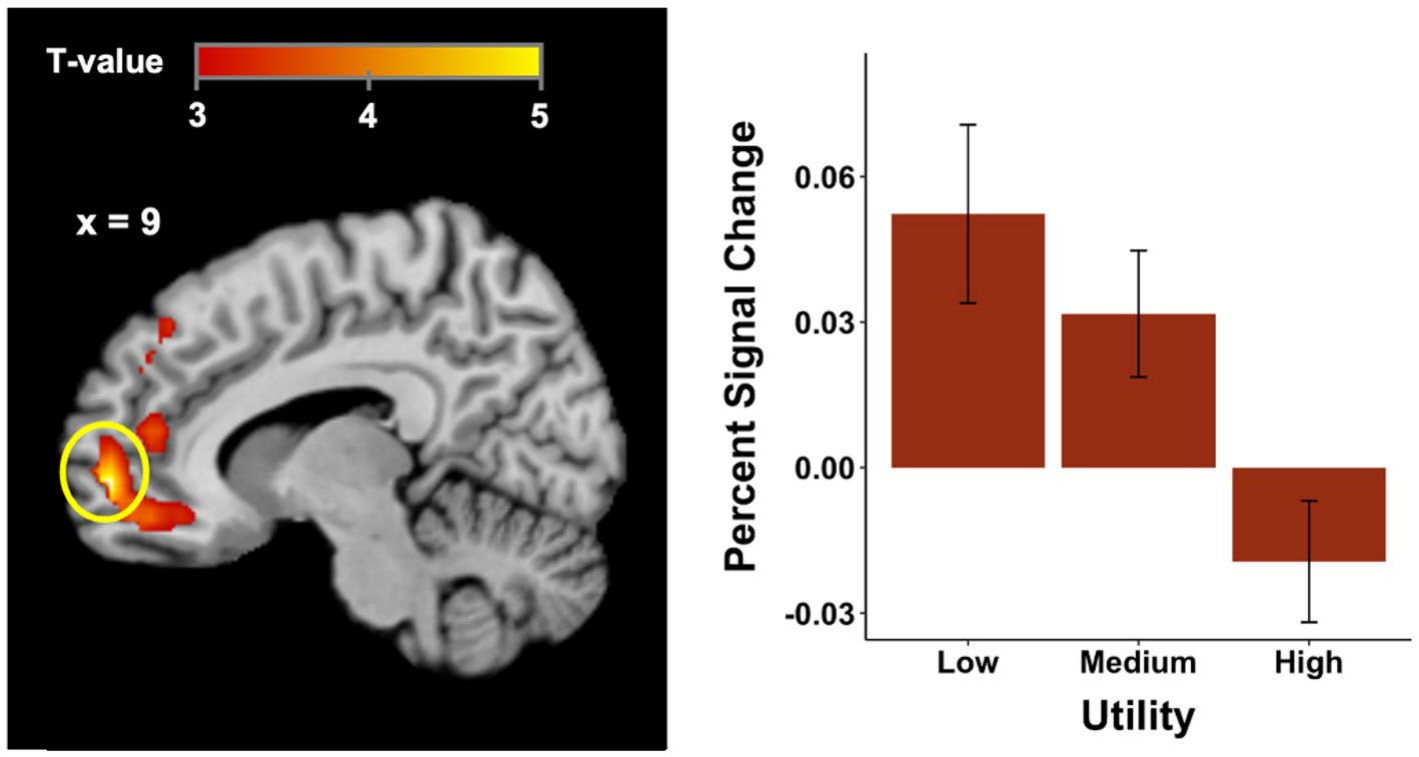
Model-based fMRI results of the utility of the punishment chosen by the third-party. The ventromedial prefrontal cortex (peak MNI coordinates 9, 57, − 3; *t*(30) = 5.08, *p*(SVC–FWE) = 0.001) was negatively correlated with the utility of the chosen punishment ($U({x}_{1},{x}_{2},{x}_{3},{p}_{SD})$) from the computational model. Percent signal changes from 3 levels of utility (Low: the lowest 1/3, Medium: the middle 1/3, High: the highest 1/3. Values from GLM2) in costly third-party punishment. The error bars show SEM.

Using GLM2, we searched for brain regions engaged with the utility of the SD-dependent chosen punishment amount. We found that only activity in the vmPFC and middle temporal gyrus correlated with the utility of the chosen punishment (see Fig. 4, left panel, and Supplemental Material Table S1). To illustrate how the BOLD signal varied with subjective utility, we extracted the percent signal change from the vmPFC (5 mm radius spheres with center at the reported peak coordinates), and found decreased BOLD signal as subjective utility increased (Fig. 4, right panel).

In addition, to visualize the extent of brain regions encoding the utility of the costly chosen punishment and inequity aversion levels, we overlapped these 2 regression analyses. The vmPFC, observed in the negative correlation with costly utility, overlapped with the vmPFC that was also observed in the positive correlation with the costly inequity level (see Supplemental Material Fig. S2). This indicates that the vmPFC integrates both inequity aversion and social distance signals but was mainly sensitive to inequity aversion to make the final decision.

## Discussion

One important feature of human social life is the prevalence of cooperative norms that guide social behavior and prescribe punishment for noncompliance^20^. Here, we combined model-based fMRI with TPP. We studied the combined effect of two factors driving sanction levels for third-party norm enforcement: (i) the social distance (SD) between the unaffected third-party and the norm violator and (ii) the inequity level between the norm violator and the recipient. It has been proposed that altruistic punishment requires three core computations: cost–benefit calculation, inequity aversion and social reference frame^21–23^. Our study provides a neurocomputational account of this proposition: the subjective value reflects a cost–benefit calculation integrating both inequity aversion and social distance, and determines the TPP decision. Our study identifies the neurocomputational mechanism underlying SD-related TPP by testing different models that compute a cost–benefit calculation that integrates the above two factors. We reveal that computation of inequity aversion and of SD between a third-party and norm violators are crucial brain mechanisms to determine a sanction during TPP.

Our behavioral findings showed that third-parties punish norm violations less when the norm violator was socially close to the participant. These findings mirror the role of SD on generosity in the vicarious reward domain^14^. In these previous studies, participants were more generous to close others, an effect referred to as social discounting. Similarly, we found that when close others violated social norms (i.e., made unfair allocations), the third-party was more tolerant (less likely to punish them). Our study confirms previous results on TPP without social distance manipulation, in which the third-party dislikes distributional inequity between the dictator and the recipient^11^. It has been proposed that the amount of punishment for crimes is driven by only two factors: the wrongdoer’s intention and the amount of harm caused to the victim^4^. However, our study indicates the need to take the strength of social ties between the wrongdoer and the punisher into consideration, and to go beyond egocentric inequity models^10^. The representation of the strength of social ties, as assessed by SD, is a key knowledge of interpersonal relationships in one’s social network^12^. These representations can be used to form social inferences and impinge on subsequent behavior including TPP. For example, primates prevent outsiders from forming alliances with their close allies, especially when this might place them at a disadvantage^24^. Our SD-dependent TPP model of inequity extends previous behavioral TPP studies that showed that outgroup perpetrators were punished more severely than ingroup perpetrators^25^. Together, our new SD-dependent TPP model of inequity incorporates both SD and the third-party perspective in the inequity model.

The brain underpinnings of altruistic punishment have previously been proposed to be composed of different brain networks^8,18^, engaged in detection and generation of an aversive experience for a social norm violation, integration of harm to the victim and intent, and inferring others’ intentions into blame. However, these processes were not captured by computational modeling. Here, we combined computational models and fMRI to address the neurocomputational mechanisms underlying norm-guided behavior.

First, our model-based fMRI findings revealed a clear overlap between the ACC and bilateral insula in inequity aversion for both SPP and TPP. A recent meta-analysis on social punishment revealed that both SPP and TPP engage the dlPFC and the bilateral anterior insula^26^. A second meta-analysis study found social punishment related activations in the bilateral insula/claustrum, the (left) superior medial frontal gyrus and the (right) inferior frontal gyrus^27^. The bilateral insula, as part of the salience network, may detect the presence or threat of norm violation and generates an aversive response, and provides an emotional measure of the severity of harm caused to the victim^8^.This brain saliency network is known to process aversive stimuli, such as empathic pain and inequity^9,28^ but also rewards^29,30^. In our study, when dictators were at higher SD, subjects were more willing to punish, and punished more severely. This indicates that these regions exhibit the capacities necessary to detect norm violation in general, and not-only as a victim. Previous meta-analyses also revealed that SPP and TPP tasks trigger different responses in the mentalizing system^27^, with TPP preferentially engaging social cognitive regions and SPP affective regions^26^. Critically, our current findings reveal that responses in the bilateral AI and rACC reflect general notions of distributional norm violation computed from others’ perspectives. Our TPP study allows us to interpret this brain system as truly reflecting inequity aversion or social norm concerns, rather than retaliatory motives because unfair offers did not affect third-parties directly^3,6^.

Second, our study determined the neurocomputational mechanisms underlying TPP when the strength of the social ties between the unaffected third-party and the wrongdoer varied. We observed a large brain network with increased activity when dictators were at greater social distances. There are different ways to define distance in social networks^31^, but it generally refers to the smallest number of ties required to connect individuals. Assessment of social distance for direct and indirect ties (friend of a friend) are important to maintain one’s reputation in a social network and to favor trust of others who share mutual friends^32^. A recent neuroimaging study characterized the network of students in an academic program, a subset of whom viewed videos of several classmates^33^. When participants viewed each classmate, network position information, including social distance, was encoded in distributed brain responses. Social distance was encoded in the inferior parietal cortex and the superior temporal cortex, consistent with the proposal that physical space around oneself and spatial distances are encoded in a similar fashion^16^. Other brain regions, including the mPFC and the hippocampus have also been proposed to encode both cognitive maps of spatial and non-spatial relational structures^34^. Thus, when encountering others, people may retrieve those individuals’ proximity to themselves according to a mental map of their social network, which may allow successful navigation in the social world. However, because participants were performing passive viewing tasks of faces or were at rest in these previous studies, it was not possible to investigate the brain computations engaged in encoding social network positions for subsequent behavior such as TPP, as we have in this model-based fMRI study.

Third, we found that the vmPFC computation reflected the subjective utility of each punishment option, consistent with its role in both individual value-based decision making^35,36^ and integration of social information^29,30,37,38^. A previous TPP study manipulated the intentionality of the norm violator and reported that vmPFC encoded the subjective value of sanctions^11^. Some studies also revealed vmPFC engagement for subjective utility of punishments^39,40^. Similarly, for decisions involving both potential gains and losses and the integration of cost–benefit, the vmPFC has been observed to reflect both appetitive and aversive values^41,42^. The vmPFC is associated with the computation of fairness by representing values of normatively valued goods^30,43^ and computes subjective value of indirect reciprocity, a type of cooperative behavior that reflects that one can transmit helping behavior to an uninvolved third person^44^. Our results therefore support that computations involving distributional inequity and strength of social ties between third-parties and norm violators are integrated to generate decisions to sanction in the vmPFC.

Cooperation between individuals seems to be at odds with evolutionary theories that individuals fight for survival and reproduction. Cooperation has clear evolutionary benefits because it favors survival of the population as a whole. Various mechanisms may explain how natural selection promotes unselfish behavior, such as TPP and indirect reciprocity^45,46^. Fundamental questions regarding the evolution of TPP remain. TPP may be crucial for punishments beyond those directly affected by norm violation^23^. Consistent with this, the level of punishment by third parties is correlated with cooperation^47^. In real life, people punish norm violators using confrontation, gossip, and social avoidance in different ways according to context^48^. That is, direct punishment (confrontation) is more likely when punishers have more to gain, for example when they have been personally victimized by norm violations. In contrast, indirect punishment (gossip and social avoidance) is more likely when the costs of potential retaliation may be large—when violations are severe and when offenders possess more relative power. Recent findings also indicate that reversing ranks and reducing inequality is more likely to occur when other’s rank/power is perceived as illegitimate, such as when high social rank is acquired through coercion or spoliation, relative to when it is acquired through merit^49^. Further studies are needed to better understand the neurocomputations required to make decisions integrating these relationships between power, norm enforcement and social distance between group members^50^. Social distance is in itself a simple measure of a highly complex construct that combines a multitude of factors including blood kinship, affection and professional relationships, to name but a few. These are very heterogeneously dispersed across different social distances and vary between different cultural groups^51,52^. Hence, larger scale studies involving greater numbers of participants will be required to disentangle the contributions of these diverse contributary factors towards tolerance or punishment of anti-social behavior.

## Materials and methods

### Participants

Thirty-four Chinese undergraduates (mean age = 20.39, SD = 1.46; 19 men) were recruited via online fliers. All participants were right-handed and had no history of psychiatric or neurological disorders. They all gave informed consent and the procedure was approved by the ethics committee of the South China Normal University (NO. 049). All experimental protocols and procedures were conducted in compliance with the latest revision of the Declaration of Helsinki. One participant was excluded from our data analysis because of random choices, two participants were excluded due to excessive head movements during scanning (> 2 mm translation or > 2° rotation), and one session of the data had to be excluded for five participants due to head movements. Finally, 31 participants mean age = 20.42, SD = 1.48 including 16 men remained.

### Procedure and tasks

#### Pre-scanning phase: social distance manipulation

On arrival, participants received verbal and written instructions for the tasks. Following the procedure by Strombach^14^, participants were first asked to rate their perceived closeness to specific persons in their social environment on a 100-point scale, i.e., mother, father, siblings, grandparents, kin, best friend, roommates, circle of friends, colleagues, neighbors, acquaintances, lover and strangers. They skipped the rating for relationships that did not exist in their social environment (e.g., lover). Before entering the scanner, participants were asked to write down one name that best corresponded to the person at the following SDs in their social entourage: 1, 2, 3, 5, 10 and 20. Notably, we also included SD levels of 50 and 100 in the fMRI experiment: 50 represented a person the participants had met but did not know well and 100 represented complete strangers. Therefore, participants were not required to indicate names for the persons at these two SDs. Furthermore, participants were explicitly asked to exclude individuals toward whom they had a negative attitude.

There were 2 practice sessions before scanning. These followed the same procedure as that during scanning, except with respect to the order of the trials. This was to familiarize participants with procedures before they entered the scanner. After completing the scanning session, participants received a 100 CYN participation payment.

#### Scanning-phase: the modified TP-DG task

**Figure 5:**
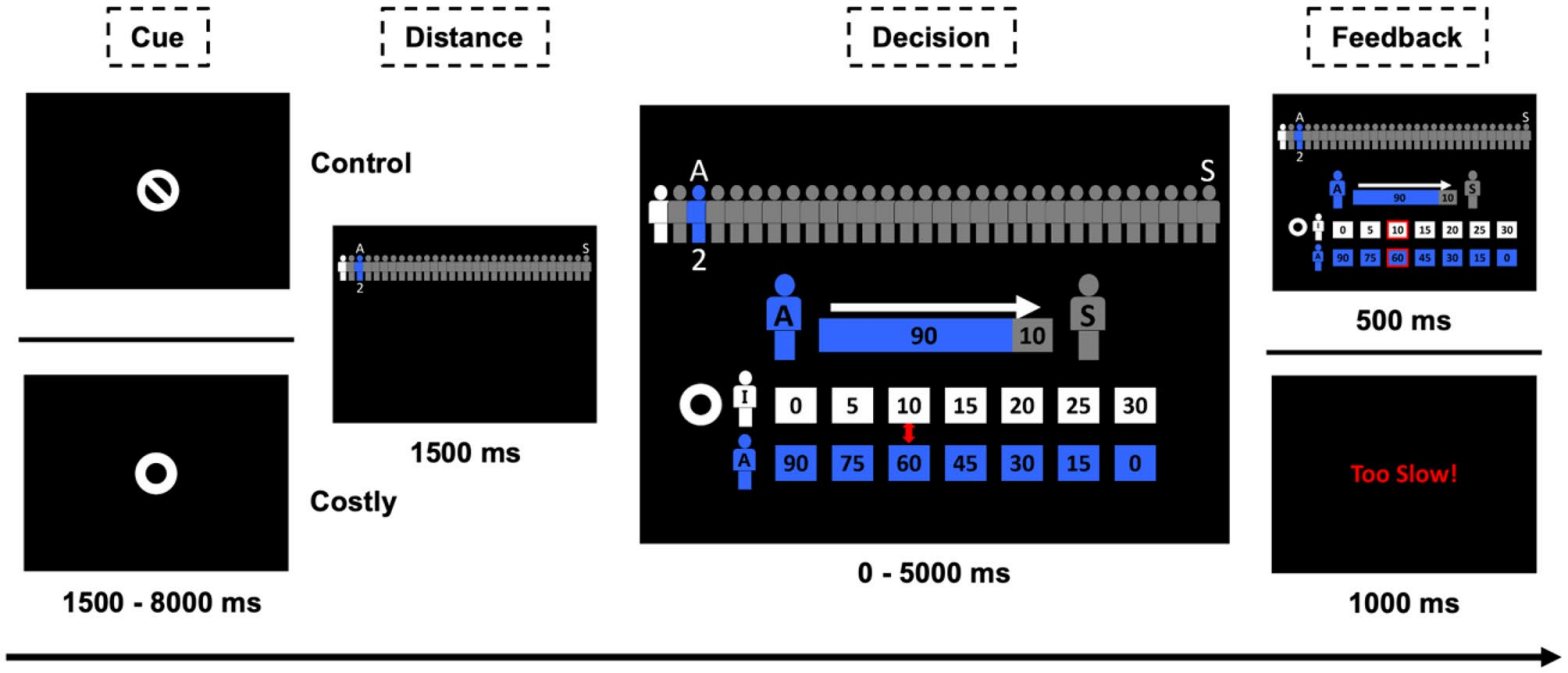
Experimental design. Participants in the scanner (represented by individual I in white on the central figure) were in the role of a third-party and could freely/costly punish norm violators (different people A in blue) while social distance between them increased. At the beginning of each block, there was an instruction screen showing the type of punishment (i.e., “free punishment” or “costly punishment”). Each trial started with a cue (a circle indicated costly punishment, a circle with a line inside indicated free punishment). Next the SD information for this trial was given on top of the screen (here only 31 icons are displayed to facilitate visualization, instead of the 101 icons shown during scanning). Then, participants were shown the unfair allocations between A and a stranger S (here 90 MU for the norm violator A and 10 for S). They were also presented with their own allocations (values in white) corresponding to each punishment options (in blue). Participants were required to choose one punishment level from 7 options, i.e., 0, 5, 10, 15, 20, 25, 30, within 5000 ms. Then, the selected option was highlighted in red as feedback (500 ms). For example, here the third-party punisher (participant) decides to use 10 MU to punish the norm violator by 30. If participants failed to make the decision in 5000 ms a warning screen (1000 ms) was shown.

We adopted a modified Third-Party Dictator Game (TP-DG)^3,5^, for the current fMRI study. Participants were instructed to consider a situation involving a dictator (labeled as player A) and a recipient (labeled as player S) (Fig. 5). The dictator was endowed with 100 CNY and could freely allocate the endowment between themself and the recipient. Decisions from the dictators could be seen by the participants inside the scanner. Participants, as third-party observers, could decide whether to use portions of their own endowment to punish unfair allocations. The key additional manipulation was for the participants to imagine that the dictators were specific members of their own social entourage that corresponded to the SD indicated between the dictator and the participant on each trial. This distinguishes the current design from a standard TP-DG in which the dictators are strangers.

The SD between the participant and the dictator was displayed iconographically on a scale consisting of 101 icons (see Fig. 5). The white icon at the left end of the scale represented the participant and the blue icon indicated a specific person A from their social entourage at social distance SD. The number under the blue icon indicated the SD between the participants and A numerically. The gray icon at the right end of the scale represented the recipient S, always at SD 100. This design allowed us to investigate the behavioral and neural effects of SD manipulation between the unaffected third-party observer and the dictator while keeping constant the SD between participants and the recipient.

Scanning was comprised of 6 sessions, each containing 54 trials. Among them, there were 48 trials displaying unfair allocations equally distributed among 12 blocks (i.e., 4 in each block). In half of the blocks, participants could punish the dictator by reducing their payoff at the cost of their own endowment (i.e., Costly condition). In the other half, they could punish the dictator without any cost to their endowment (i.e., Free punishment condition, reflecting the control condition). The target trials covered all 24 combinations between SDs (i.e., 1, 2, 3, 5, 10, 20, 50, 100) and unfair allocations (i.e., 90 vs. 10, 85 vs. 15, 80 vs. 20), with each combination appearing once for each condition respectively, the order of the trials were pseudorandomized. Furthermore, we added 6 filler trials displaying quasi-fair allocations (i.e., 65 vs. 35, 60 vs. 40, 55 vs. 45), randomly assigned to the 6 blocks. These quasi-fair trials were included because in a pilot study we found that when all the allocations were unfair participants accepted the unfair allocations as “normal behavior” and did not punish. We decided to include quasi-fair trials, as opposed to completely fair trials (50/50 split), both to reflect the difference in the power of dictators and the recipients but also so that these trials would not stand out so obviously from the unfair allocations in the other trials. All blocks and trials were presented pseudo-randomly.

Each block began with a 3000 ms notification of the punishment condition (see Fig. 5). In each trial, participants were endowed with 50 CNY. The trial started with a cue to indicate the punishment type (i.e., Costly or Free), which lasted for a jittered interval (between 3000 and 8000 ms). Next, a 1500 ms screen with the SD information was displayed. This was followed by the decision screen on which participants saw the money allocation made by the dictator to the stranger, and were provided with options of different punishment levels (0, 5, 10, 15, 20, 25, 30 CNY). The dictators would be punished three times as much as the chosen punishment option. For example, the allocation of 90 vs 10 could result in a payoff of 90, 75, 60, 45, 30, 15, or 0 CNY for the dictator, depending on the degree of punishment chosen by the participant. Participants were required to select one option within 5000 ms, by pressing two buttons to move the cursor (with a random initial position), and confirmed the final choice by pressing another button with their right hand. Participants were required not to move the cursor until they determined the final option. Once they confirmed their choice, a red frame appeared on the chosen option for 500 ms. If the participants confirmed their decision within 5000 ms, the jittered cue of the next trial would show, and if they failed to respond within 5000 ms, a warning screen was shown for 1000 ms (see details in Fig. 5).

#### Behavioral data analysis

All behavioral analyses were conducted using R (http://www.r-project.org/) and relevant packages. All the reported p values are two-tailed and *p* < 0.05 was considered to be statistically significant. Data visualization was performed via ‘ggplot2’ package (https://ggplot2.tidyverse.org).

Regarding the punishment amount data, we performed a mixed-effect linear regression model on the punishment amount using the lmer function in ‘lme4’ package (http://cran.r-project.org/package=lme4), with payment conditions (Costly/Control), social distances (as a continuous variable), inequity levels (90 vs. 10/85 vs. 15/80 vs. 20), and both 2-way and 3-way interactions as the fixed-effect predictors. In addition, we included a random intercept and random by-subject slopes for the three factors and their interactions per participant. For the statistical inference on each predictor, we performed a Type III ANOVA with Satterthwaite’s method on the model fits by using the anova function. Post-hoc multiple comparisons were conducted using the emmeans function in ‘emmeans’ package (http://cran.r-project.org/package=emmeans).

For reaction time (RT), we also performed a mixed-effect linear regression model on RT by the lmer function, with payment conditions (costly/control), social distances (as a continuous variable), inequity levels (90 vs. 10/85 vs. 15/80 vs. 20), and both 2-way and 3-way interactions as the fixed-effect predictors, random-effect factors were specified in the same way as above. The statistical inference on each predictor and the post-hoc multiple comparisons were conducted in the same way as above.

We also built computational models to further understand the decision making process. The model estimation and comparison were performed using MATLAB (Mathworks Inc., Sherbom, MA). and the VBA toolbox^53^.

#### Computational model of the effect of social distance on third-party punishment and estimation procedures

To investigate the neurocomputational mechanisms underlying the effects of SD on TPP, we developed a new computational model based on a study of TPP that shows that individuals assign values to all of the options, and then compare their computed values to select one of them. A classic inequity aversion model assumed that people felt inequity either when they were worse or better off than other players, and suffer more from inequity when they are in disadvantaged than when they are advantaged^10^. However in TPP, participants are concerned by the inequity between the dictator and the recipient, for this reason a third-party inequality aversion model (called TPIA model; Eq. 1) was developed^11^. This model estimates the subjective utility of the observer for a given level of punishment and a level of inequity between the dictator and the recipient. We assumed that the observer dislikes the distributional inequity between the dictator and the recipient. As punishment is costly, the observer is required to trade-off between their own payoff and the level of distributional inequity between the dictator and the recipient as follows:

1 $$U\left({x}_{1},{x}_{2},{x}_{3},p\right)=\mathrm{max}({x}_{3}-p,0)-\gamma \times abs\left(\mathrm{max}({x}_{1}-3p,0)- {x}_{2}\right)$$

Equation (1) shows the other-regarding third-party inequality aversion model. $U$ is the subjective utility of the observer (i.e., third-party decision maker) for a given amount of punishment $p$. ${x}_{1},{x}_{2},{x}_{3}$ represent the initial material payoff of the dictator, the recipient, and the observer, respectively. With ${x}_{3}$ being always equal to 50. ${x}_{3}-p$ represents the earnings of the observer, given a certain level of punishment, and cannot be lower than 0, and $abs\left(\mathrm{max}({x}_{1}-3p,0)- {x}_{2}\right)$ represents the difference in allocation between the dictator (in the current study we manipulated that it cannot be lower than 0) and the recipient after punishment. Finally, $\gamma$ describes the degree of inequity aversion caused by the difference in allocations between the dictator and the recipient (0 ≤ $\gamma$  ≤ 1). Subjects would compute the overall utility for all the seven punishment options and choose the option with the highest utility.

Based on this initial TPIA model, we tested a number of functions (hyperbolic discounting and flexible power functions) to account for the relationship between SD and punishment behavior. We tested four potential candidates, the first and second were based on the hyperbolic discount function^13^. We either applied this function on the level of punishment (Hyperbolic punishment model) or the degree of inequity aversion (Hyperbolic inequity model). In the case of punishment discounting, we considered that the observers would increase their level of punishment as the SD between them and the dictator increased. Formally, the chosen punishment level ${p}_{SD}$ is transformed into the subjective level of punishment $p$ as follows:

2 $$p=\left(1+\frac{1}{k\times SD}\right)\times {p}_{SD}$$

With $k$ being the discounting rate and $SD$ the social distance. When considering the degree of inequity aversion $\gamma$, the logic is reversed as the higher the SD, the lower discounting of inequity aversion should be. Accordingly, we applied the hyperbolic discounting function directly on $\gamma$. Formally, the initial inequity aversion ${\gamma }_{SD}$ is transformed into the discounting inequity aversion $\gamma$ as follows:

3 $$\gamma =Softmax\left(\left(1+\frac{1}{k\times SD}\right)\times {\gamma }_{SD}\right)$$

As $\gamma$ is constrained to be between 0 and 1, we applied a softmax function on the hyperbolic discounting function and let ${\gamma }_{SD}$ takes any value.

For the last two candidates, instead of using the hyperbolic discounting function, we tested the flexible power function^54^. In this function, punishment (inequity aversion) is inflated by an increasing amount as SD grows. Formally, for punishment the function is as follows (Power punishment model):

4 $$p={p}_{SD}+k\times S{D}^{W}$$

With $k$ being the curvature of the power function, $SD$ the social distance and $W$ the power level. The equation for the inequity aversion parameter is (Power inequity model):

5 $$\gamma =Softmax({\gamma }_{SD}+k\times S{D}^{W})$$

We estimated each of the four models using the VBA Toolbox in Matlab. A Bayesian Model Selection (BMS) was performed using the same toolbox in a random effect analysis relying on the free energy as the lower bound of model evidence. We used protected Exceedance Probability measurements (pEP) to select the model which was used most frequently in our population^55^. Our results show that the Hyperbolic punishment model (Eq. 2), achieving a pEP of 0.999, outperformed every other model. With this winning model, we combined it with the third-party inequality aversion model (TPIA model; Eq. 1), and then computed the utility of the chosen level of punishment for each decision by each participant and used this as the main parameter in the following fMRI analysis.

To better illustrate the relationships between the calculated utility from the winning model and our manipulated variables, we plotted a color-coded heatmap of the utility as a function of the social distance and different inequity levels (see Fig. 1B). The observed pattern showed that increased social distance between the third-party and the dictator was associated with increased utility of the chosen punishment, but only when social distance remained below 50. This might be due to the fact that the third-party only had real relationships with dictators at social distances 1 to 20, while dictators at social distance 50 and 100 were unknown to the third-party. We also observed that utility decreases with higher inequity levels.

#### fMRI scanning parameters and data preprocessing

Scanning was performed on a 3-T Trio Scanner (Siemens). Functional data were acquired using echo-planar imaging sequences (repetition time = 2 s, echo time = 30 ms, flip angle = 90°, field of view = 224 mm, slice thickness = 3.5 mm). A total of 32 axial slices were acquired in interleaved order (in-plane resolution 3 × 3 mm). Anatomical images were T1-weighted (MDEFT, 1 × 1 × 1 mm resolution). The presentation of the task and recording of behavioral responses were performed using E-Prime 2.0 software (https://pstnet.com/products/e-prime/). Neural data of 34 participants were analyzed using SPM12 (http://www.fil.ion.ucl.ac.uk/spm/software/spm12/) implemented in MATLAB 7.8 (Mathworks Inc., Sherbom, MA). The results are visualized using Mango software (Lancaster, Martinez; http://www.ric.uthscsa.edu/mango).

Functional images were realigned using a six-parameter rigid-body transformation. Each individual’s structural T1 image was co-registered to the average of the motion-corrected images using 12-parameter affine transformation. Individual T1 structural images were segmented into grey matter, white matter, and cerebrospinal fluid. Functional images were, in order, slice timing corrected, motion corrected, segmented using the T1-weighted image, normalized to MNI space, and smoothed with an 8 mm isotropic Gaussian kernel.

#### fMRI data analysis

We constructed two general linear models (GLM) to explore the decision process. In the first GLM (GLM 1), for each participant, a first-level intra-individual analysis was conducted with six regressors of interest per session: a regressor modeling the decision phase of control trials, with two parametric regressors modeling the social distance and inequity level on each trial, and the same regressors for costly trials. In GLM 2, we constructed the fMRI design matrix with three regressors of interest per session: a regressor modeling the decision making onset of control trials, a regressor modeling the decision making onset of costly trials, with a parametric regressor modeling the trial-wise chosen utility on each trial. All the events were modeled as stick functions with duration zero.

We included six additional event regressors of no interest, describing the onsets of: (i) The verbal instruction at the beginning of each block; (ii) The SD information of each trial; (iii) The punishment options for filler trials; (iv) The punishment options for no-response trials; (v) The feedback for responded trials; (vi) The feedback for no-response trials. These events were all modeled as stick functions with duration zero. Finally, six motion regressors obtained during realignment were included to control for motion of no interest.

We implemented standard general linear models (GLMs) for model-based univariate fMRI analysis. First-level analyses were conducted using fixed-effect models. Second-level analyses were conducted using random-effect models in SPM12. All images were high-pass filtered in the temporal domain (filter width 128 s). Autocorrelation of the hemodynamic responses were modeled as an AR(1) process.

For small-volume correction analysis, we used ACC, PCC, and IPL atlases from automated anatomical atlas (aal) template, and spheres of 12-mm centered on coordinates from previous meta-analyses. More specifically, we used coordinates of vmPFC (MNI coordinate, x = 0, y = 52, z = − 8) from a meta-analysis where activities in the region were correlated with subjective value for monetary incentives^56^. We adopted coordinates of bilateral anterior insula (MNI coordinate, x = − 34, y = 18, z = − 12 and x = 34, y = 16, z = − 18) from a meta-analysis where activities in these regions were correlated with the Trust (TG) and the Ultimatum game (UG)^18^.

## Supplementary Information

Supplementary Information.

## Data Availability

All the raw data for the current study are available in an open data repository (https://github.com/zixtang/Social_Punishment_fMRI, it is a private repository, and we will make it public upon acceptance of the paper).
