# Supplementary material for: Strengths of social ties modulate brain computations for third-party punishment
Source: Sci Rep. 2023 Jun 28;13:10510. doi: 10.1038/s41598-023-37286-8 (PMC10307782; doi:10.1038/s41598-023-37286-8)
Supplement: Supplementary file 1 — Supplementary Information. [file 41598_2023_37286_MOESM1_ESM.docx]

**Supplementary Information for**

How strength of social ties modulates brain computations for Third-Party Punishment

Zixuan Tang^a^, Chen Qu^a^, Yang Hu, Julien Benistant, Frédéric Moisan, Edmund Derrington, Jean-Claude Dreher*

^a^These authors contributed equally to this work.

*Correspondence to: [dreher@isc.cnrs.fr](mailto:dreher@isc.cnrs.fr)


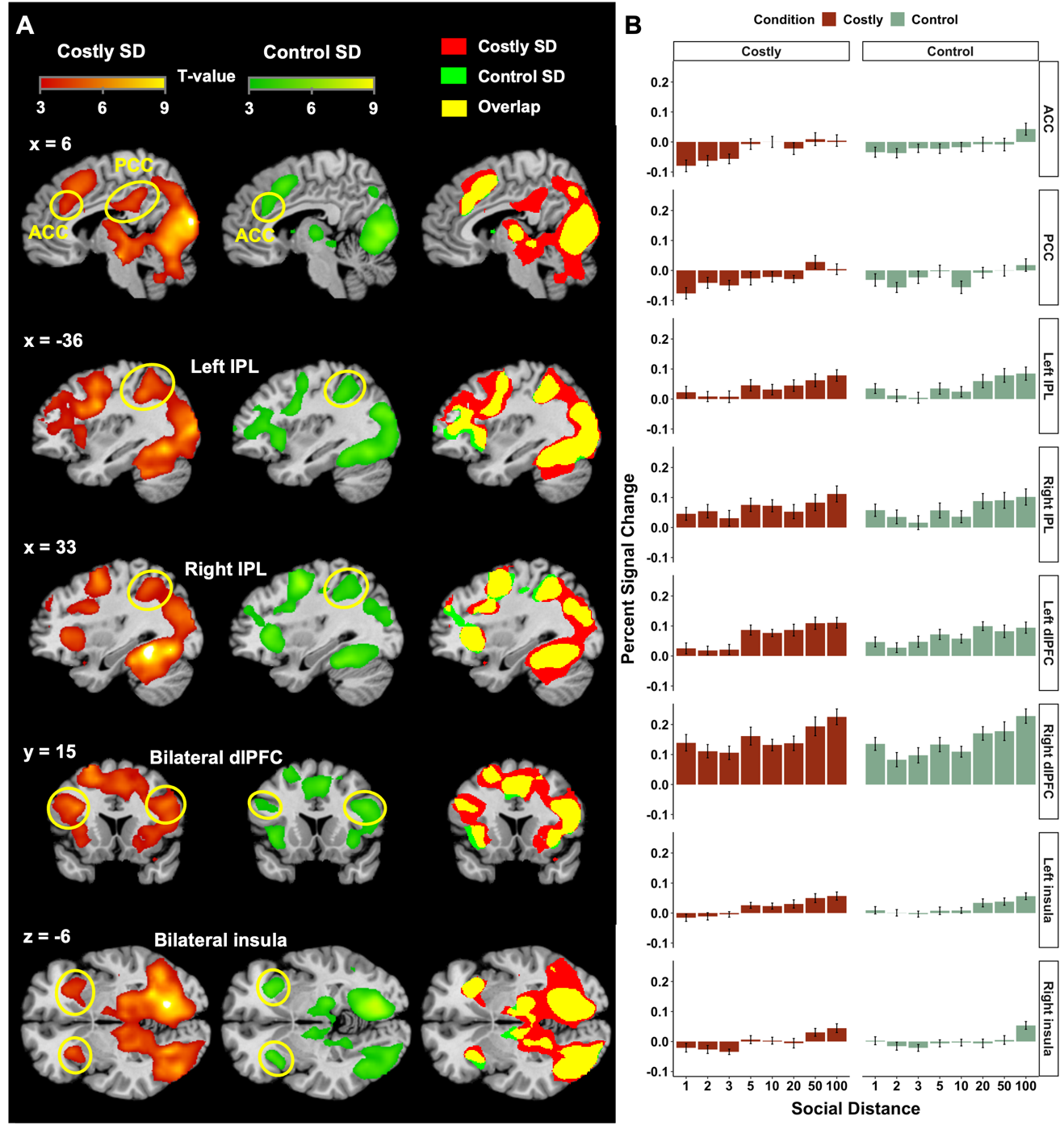


Figure S1. Overlay of costly and control Social Distance (SD) fMRI results (GLM 1). (A) Brain regions correlating positively with increasing SD in Costly condition and Control. (B) The percent signal change from the indicated brain regions (5 mm radius sphere centered at peak coordinates) were extracted for social distance in the Costly and Control conditions. The error bars show SEM.


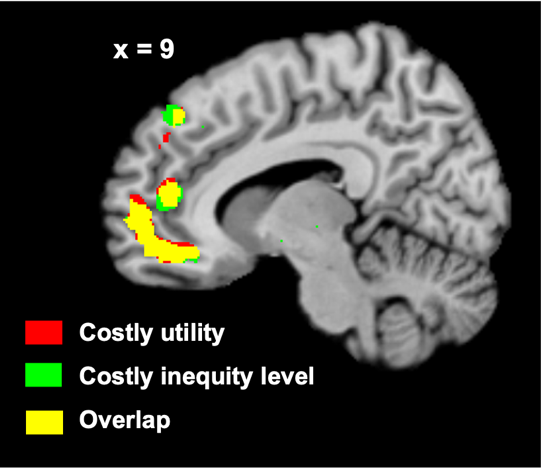


**Figure S2.** Overlay of brain activation for Costly utility and Costly inequity level showing overlap vmPFC activation.

**Table S1.** Brain regions involved in social distance and the utility of the chosen punishment related activity. Coordinates x, y, z (mm) are given in Montreal Neurological Institute (MNI) standard stereotactic space. All results are reported at the voxel level *p*(FWE) < .05 corrected.

| Regions | Cluster size (*k*) |  | Voxel-level statistics | |  | MNI coor. | | |  |
| --- | --- | --- | --- | --- | --- | --- | --- | --- | --- |
|  |  |  | *T*-val | *p*_FWE_ |  | x | y | z |  |
| **Positively correlated with Social Distance** | | | | | | | | |  |
| R Fusiform gyrus | 10155 |  | 12.51 | < 0.001 |  | 27 | -51 | -12 |  |
| R Cuneus | 10155 |  | 11.99 | < 0.001 |  | 9 | -90 | 15 |  |
| R Lingual gyrus | 10155 |  | 10.07 | < 0.001 |  | 15 | -66 | -9 |  |
| R Calcarine cortex | 10155 |  | 9.20 | < 0.001 |  | 12 | -75 | 6 |  |
| L Precuneus | 10155 |  | 9.15 | < 0.001 |  | -15 | -66 | 30 |  |
| L Fusiform gyrus | 10155 |  | 8.67 | < 0.001 |  | -27 | -66 | -12 |  |
| L Middle occipital gyrus | 10155 |  | 8.67 | < 0.001 |  | -27 | -81 | 24 |  |
| R Inferior temporal gyrus | 10155 |  | 8.15 | < 0.001 |  | 48 | -57 | -9 |  |
| R Middle occipital gyrus | 10155 |  | 8.13 | < 0.001 |  | 27 | -90 | 18 |  |
| R Middle temporal gyrus | 10155 |  | 7.97 | < 0.001 |  | 42 | -69 | 3 |  |
| L Precentral gyrus | 3626 |  | 7.81 | < 0.001 |  | -42 | 3 | 27 |  |
| L Middle frontal gyrus | 3626 |  | 7.15 | 0.001 |  | -24 | 12 | 63 |  |
| R Dorsal lateral prefrontal cortex | 3626 |  | 6.58 | 0.005 |  | 42 | 9 | 24 |  |
| R Precentral gyrus | 3626 |  | 6.58 | 0.005 |  | 30 | -3 | 51 |  |
| L Dorsal lateral prefrontal cortex | 3626 |  | 6.37 | 0.008 |  | -36 | 15 | 24 |  |
| L Superior frontal gyrus | 3626 |  | 6.30 | 0.010 |  | -18 | 15 | 51 |  |
| **Negatively correlated with the Utility** | | | | | | | | |  |
| R Middle temporal gyrus | 160 |  | 7.59 | 0.001 |  | 54 | -3 | -24 |  |
